# ANAC005 is a membrane‐associated transcription factor and regulates vascular development in *Arabidopsis*

**DOI:** 10.1111/jipb.12379

**Published:** 2015-09-25

**Authors:** Jun Zhao, Jiang‐Shu Liu, Fu‐Ning Meng, Zhen‐Zhen Zhang, Hao Long, Wen‐Hui Lin, Xiao‐Min Luo, Zhi‐Yong Wang, Sheng‐Wei Zhu

**Affiliations:** ^1^ Key Laboratory of Plant Molecular PhysiologyInstitute of Botany, Chinese Academy of SciencesBeijing100093China; ^2^ University of Chinese Academy of SciencesBeijing100049China; ^3^ Institute of Molecular Cell Biology, College of Life ScienceHebei Normal UniversityShijiazhuang050016China; ^4^ Department of Plant BiologyCarnegie Institution for ScienceStanfordCalifornia94305USA

**Keywords:** ANAC005, Arabidopsis, NAC, vascular tissues development

## Abstract

Vascular tissues are very important for providing both mechanical strength and long‐distance transport. The molecular mechanisms of regulation of vascular tissue development are still not fully understood. In this study we identified ANAC005 as a membrane‐associated NAC family transcription factor that regulates vascular tissue development. Reporter gene assays showed that ANAC005 was expressed mainly in the vascular tissues. Increased expression of ANAC005 protein in transgenic *Arabidopsis* caused dwarf phenotype, reduced xylem differentiation, decreased lignin content, repression of a lignin biosynthetic gene and genes related to cambium and primary wall, but activation of genes related to the secondary wall. Expression of a dominant repressor fusion of ANAC005 had overall the opposite effects on vascular tissue differentiation and lignin synthetic gene expression. The ANAC005‐GFP fusion protein was localized at the plasma membrane, whereas deletion of the last 20 amino acids, which are mostly basic, caused its nuclear localization. These results indicate that ANAC005 is a cell membrane‐associated transcription factor that inhibits xylem tissue development in *Arabidopsis*.

**Edited by**: Jan Traas, University of Lyon, France

## INTRODUCTION

About 2,500 transcription factors (TFs) have been identified in the *Arabidopsis* genome (Perez‐Rodriguez et al. 2010). These include about 105 members of the NAC family of plant‐specific transcription factors, which are named for NAM (No apical meristem), ATAF (*Arabidopsis* transcription activation factor) and CUC (Cup‐shaped cotyledon) (Ooka et al. 2003). NAC proteins contain a conserved N‐terminal NAC domain and a C‐terminal transcription regulatory region (Ernst et al. 2004; Olsen et al. 2005). A typical NAC domain can be divided into five subdomains: the C and D subdomains are highly conserved and responsible for DNA binding; the A subdomain may be involved in the formation of homodimer or heterodimer; the B and E subdomains may be responsible for the functional diversity of NAC genes (Ooka et al. 2003; Ernst et al. 2004; Jensen et al. 2010; Chen et al. 2011). However, several atypical NAC proteins have been identified, including proteins with the NAC domain only (Christiansen et al. 2011), some NAC proteins containing two tandemly repeated NAC domains (Jensen et al. 2010), and NAC proteins containing a zinc finger domain to the N‐terminal of NAC domain (Mitsuda et al. 2004; Jensen et al. 2010).

The activity of transcription factors can be regulated by several processes, including posttranscriptional modification, protein‐protein interactions, and nuclear transport (Poon and Jans 2005; Espenshade and Hughes 2007). Many transcription factors have been found physically tethered to the membrane and released by proteolytic cleavage (Hoppe et al. 2001; Liu et al. 2007a, 2007b; Iwata et al. 2009). A genome‐wide analysis predicted 85 membrane‐associated transcription factors (MTFs) in *Arabidopsis* (Kim et al. 2010). These include 18 NAC proteins predicated to contain α‐helical transmembrane motifs (TMs) in the C‐terminal region that may mediate association with the cell membrane, and they are named NAC with Transmembrane Motif 1 (NTM1), and NTM1‐like (NTL) factors (Kim et al. 2007b). It is notable that virtually all of the reported plant MTFs are closely related with plant responses to various abiotic stress conditions. NTM1/NTL12 and NTM2/NTL13 are released from membrane by proteolytic cleavage in response to salt treatment (Kim et al. 2006; Park et al. 2011). A truncated NTM1 that is unable to associate with membrane causes reduced cell numbers and defects in cell division. Membrane association of NTL8 is induced by salinity and GA deficiency, and NTL8 inhibits seed germination and flowering (Kim et al. 2007a, 2008). The processing of NTL9 is promoted by osmotic stress, and NTL9 in turn regulates some senescence‐associated genes (Lim et al. 2007; Yoon et al. 2008). Release of NTL6 from the membrane is activated by cold, drought, high salinity and abscisic acid (ABA) (Clarke et al. 2004; Kim et al. 2007b). Interestingly, pathogenesis‐related genes were significantly induced in these transgenic plants, suggesting that NTL6 might be an integrator of biotic and abiotic stress responses (Seo et al. 2010). These studies suggest that plant MTFs are closely related with plant responses to various abiotic stress conditions (Kim et al. 2010).

Several NAC family proteins have been shown to be involved in the regulation of vascular development. *SND1* (secondary wall‐associated NAC domain protein 1) is a NAC family gene that is specially expressed in interfascicular fibers and xylary fibers. Ectopic overexpression of *SND1* induces the expression of several secondary wall biosynthetic genes (such as *CESA7* and *CESA8*) and the thickening of secondary wall. A dominant repressor form of SND1 reduces the thickness of secondary wall (Zhong et al. 2006, 2007). NST1 (NAC secondary wall thickening promoting factor 1), NST2 and NST3 are three homologous NAC proteins that activate secondary cell wall formation redundantly (Mitsuda et al. 2007, 2005; Zhong et al. 2007; Mitsuda and Ohme‐Takagi 2008). Similar to SND1, overexpression of NST1 and NST3 induces the thickening of a secondary cell wall in aboveground tissues. Expression of dominant negative forms of NST1 and NST3 repress the cell wall thickening in interfascicular fiber tissue independently. The cell wall thickening of presumptive interfascicular fiber tissue is also suppressed in double mutant of *nst1‐1 nst3‐1* (Mitsuda et al. 2007; Zhong et al. 2007; Mitsuda and Ohme‐Takagi 2008). NST1 and NST2 redundantly activate secondary cell wall formation in endothecium of anther (Mitsuda et al. 2005). VASCULAR‐RELATED NAC‐DOMAIN proteins belong to a subfamily of NACs that includes seven members. VND6 and VND7 play an important role in the regulation of metaxylem vessel and protoxylem formation (Kubo et al. 2005; Yamaguchi et al. 2010, 2008, 2011). VNI2 is another NAC protein that interacts with VND7 in yeast two‐hybrid screen. VNI2 inhibits the formation of xylem vessel through reducing the expression of secondary wall related genes that are regulated by VND7 (Yamaguchi et al. 2010).

In this study, we studied the function of *ANAC005*. We found that *ANAC005* is expressed preferentially in the vascular tissue, and increasing the expression of ANAC005 proteins caused oval‐shaped leaves and short petioles, and reduced xylem tissue differentiation, whereas a dominant repressor form of ANAC005 enhanced xylem tissue differentiation, accompanied with altered expression of genes involved in vascular tissue development. While ANAC005 was not predicted to be an MTF, it is localized at the cell membrane through its C‐terminal domain, which is highly basic. Our results indicate that ANAC005 is a membrane‐associated NAC family transcription factor that plays an important role in vascular development in *Arabidopsis*. This study also identifies a novel motif that mediates membrane association of transcription factor.

## RESULTS

### *ANAC005* is predominantly expressed in xylem

**Figure 1:**
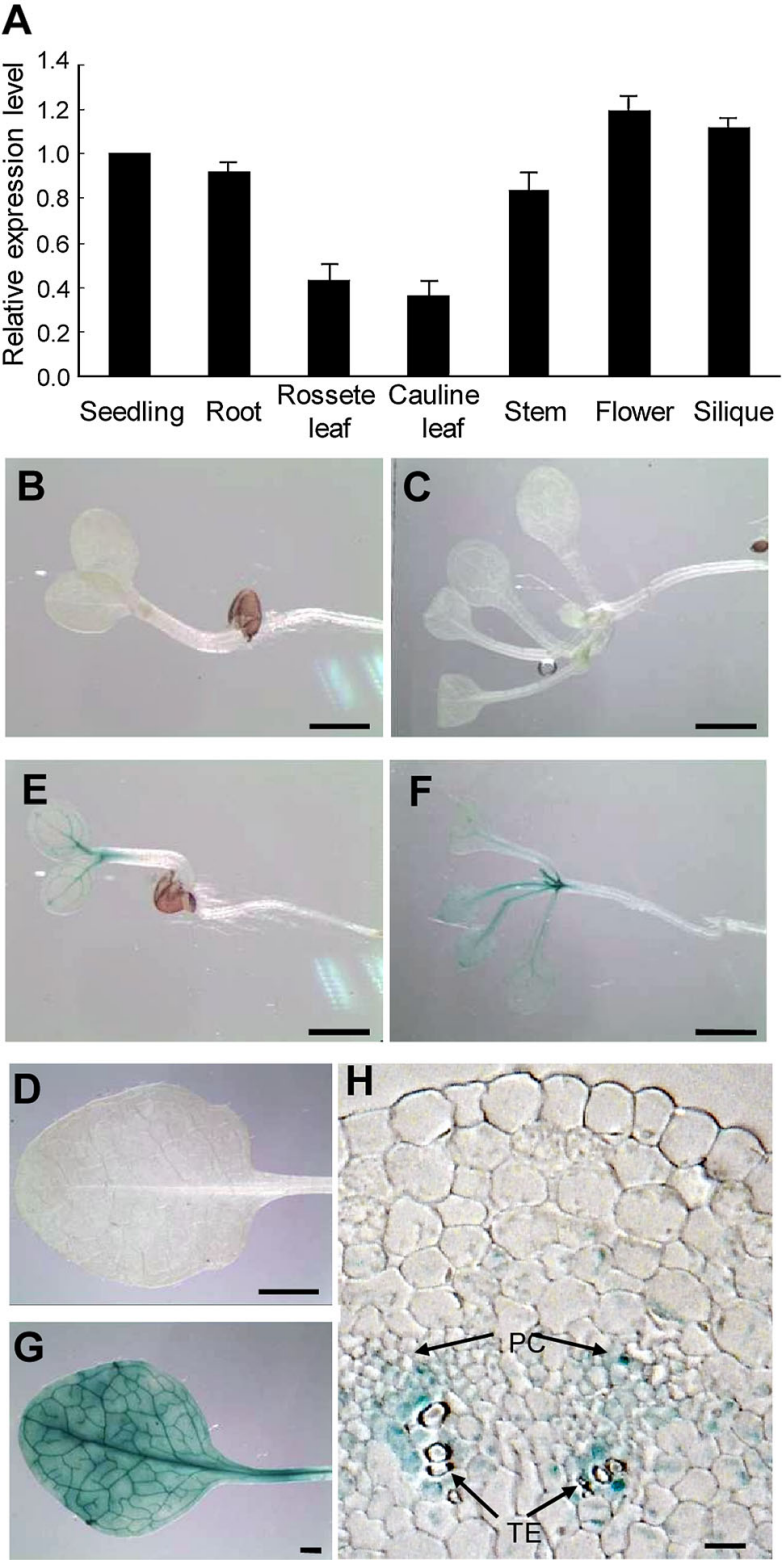
***ANAC005* is expressed preferentially in vascular bundles** **(A)** Analysis of *ANAC005* expression level in different organs by quantitative reverse transcription‐polymerase chain reaction (qRT‐PCR). The expression level in the seedling is set to 1.0, and error bars represent SD of three biological replicates. **(B**–**H)** GUS staining of wild type and *ProANAC005:ANAC005‐GUS* plants. **(B)** 3‐day‐old wild type seedling. **(C)** 10‐day‐old wild type seedling. **(D)** 3‐day‐old *ProANAC005:ANAC005‐GUS* seedling. **(E)** 10‐day‐old *ProANAC005:ANAC005‐GUS* seedling. **(F)** rosette leaf of *ProANAC005:ANAC005‐GUS* plants. **(G)** Close‐up image of cotyledon from 5‐day‐old *ProANAC005:ANAC005‐GUS* seedling. **(H)** transverse section of inflorescence stem from 5‐week‐old *ProANAC005:ANAC005‐GUS* plants. PC, phloem cell; TE, tracheary element. Scale bars = 1 mm in **(B–F)**, 20 µm in **(G)** and **(H)**.

ANAC005 is a NAC gene preferentially expressed in xylem according to a tissue specific microarray analysis (Zhao et al. 2005). We first performed quantitative reverse transcription‐polymerase chain reaction (qRT‐PCR) to examine the expression of ANAC005 in different *Arabidopsis* tissues and organs. *ANAC005* was ubiquitously expressed at a similar level in 1‐week‐old seedlings, roots and rosette leaves of 3‐week‐old plants, stem of 4‐week‐old plants and other organs of 6‐week‐old plants (Figure 1A). To further investigate the expression pattern of *ANAC005*, a 2.7 kb genomic sequence, including 1.1 kb promoter region and the 1.6 kb coding region, was fused to the *Escherichia coli* β‐glucuronidase (GUS) reporter gene and transformed into *Arabidopsis*. As shown in Figure 1B–H, the *proANAC005::ANAC005‐GUS* transgenic plants showed GUS activity in the vascular system of the cotyledons, rosette leaves, petioles, and inflorescence stem. A section of the inflorescence stem shows the highest GUS signal in the thin‐walled cells (presumably developing tracheary element) adjacent to the thick‐walled tracheary elements in xylem. No GUS signal was observed in the mature tracheary elements, suggesting that ANAC005 is highly expressed during tracheary element development. Weak GUS signals were also observed in the phloem cells of vascular tissue (Figure 1H). GUS expression was also detected preferentially in vascular tissues of cauline leaves, petals, and stigma. These results indicate that ANAC005 is mainly expressed in the vascular tissues of all organs in *Arabidopsis*. These results are consistent with the reported microarray result showing that *ANAC005* is expressed at a higher level in xylem compared with phloem and non‐vascular system (Zhao et al 2005).

### ANAC005 localizes to the cell membrane through its C‐terminus

**Figure 2:**
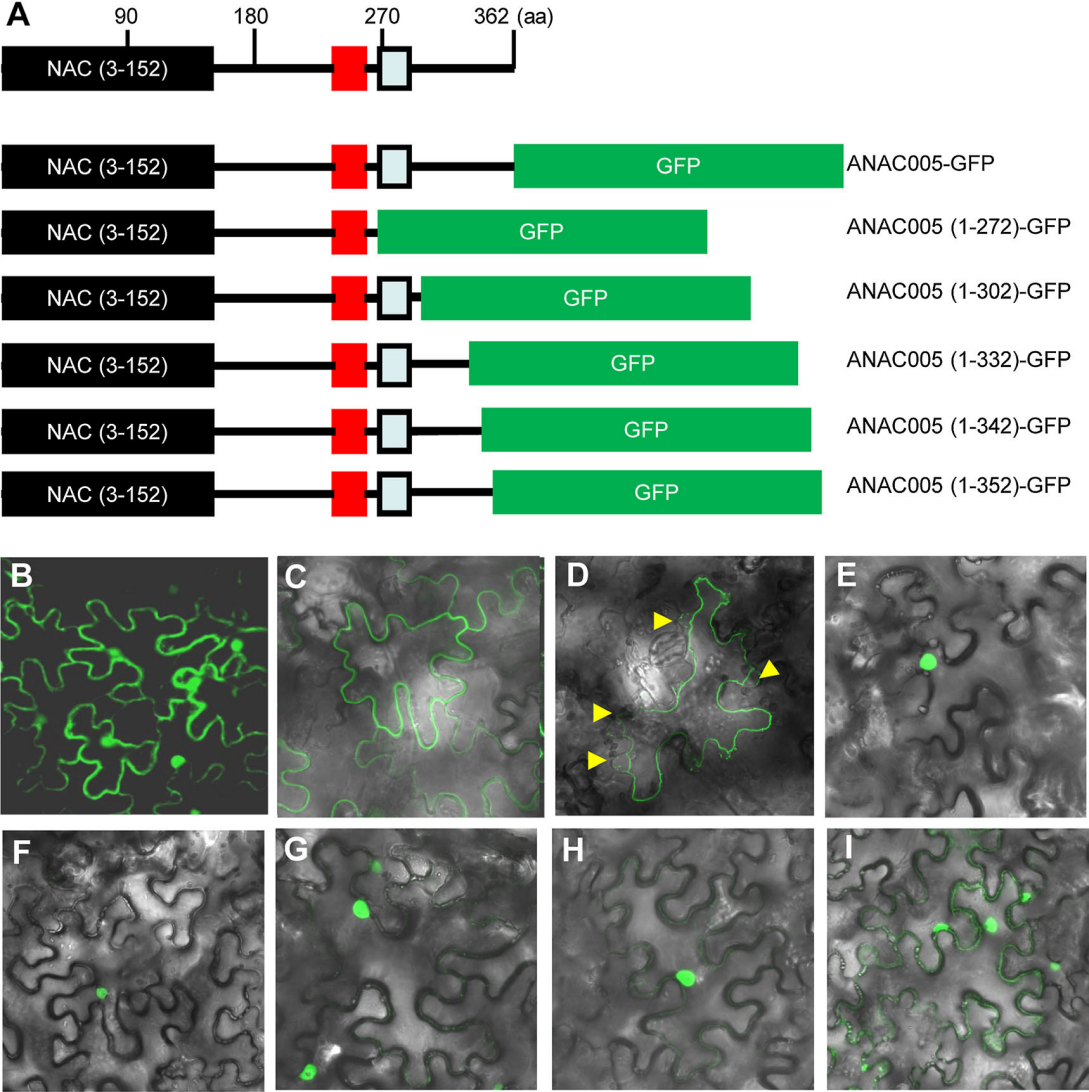
**Subcellular localization of ANAC005‐green fluorescent protein (GFP) protein** **(A)** Diagram of ANAC005 protein and GFP fusion proteins. Red box shows transcription activation domain; blue box shows putative transmembrane domain. **(B, C, E−I)** Fluorescence microscopic image of *Nicotiana benthamiana* leaf epidermal cells transformed with *35S:GFP* **(B)**, *35S:ANAC005‐GFP* **(C)**, *35S:ANAC005(1‐272)‐GFP* **(E)**, *35S:ANAC005(1‐302)‐GFP* **(F)**, *35S:ANAC005(1‐332)‐GFP* **(G)**, *35S:ANAC005(1‐342)‐GFP* **(H)** and *35S:ANAC005(1‐352)‐GFP* **(I)**. **(D)** Fluorescence microscopic image of plasmolysis of *Nicotiana benthamiana* leaf epidermal cells transformed with *35S:ANAC005‐GFP*. Yellow arrows marker the places where plasmolysis happen.

To determine the subcellular localization of ANAC005, we made an ANAC005‐GFP (green fluorescent protein) fusion protein construct, driven by the Cauliflower mosaic virus (*CaMV)* 35S promoter, and transformed it into *Nicotiana benthamiana*. In the epidermal cells of *Nicotiana benthamiana* transformed with *35S::GFP*, GFP fluorescence was visible in both nucleus and cytoplasm (Figure 2B). By contrast, the ANAC005‐GFP fluorescence was only detected at the plasma membrane (Figure 2C, D). Protein structure analysis showed that the N‐terminal of ANAC005 has a NAM domain including A, B, C, D and E motifs; its C‐terminus is a putative transcription regulation domain (Figure S1). ANAC005 does not belong to the 18 NAC proteins previously identified as putatively MTFs. We further examined the ANAC005 sequence using the TopPred‐v2 program, and identified a putative transmembrane motif (TM motif) in the middle region (aa268‐288). In order to test whether the putative TM motif determines the localization of ANAC005, we made several truncated ANAC005 proteins fused with GFP protein (Figure 2E–I). When expressed in *Nicotiana benthamiana* driven by the CaMV 35S promoter, full‐length ANAC005‐GFP was localized exclusively at the plasma membrane (Figure 2C). In contrast, ANAC005(1‐272)‐GFP (with deletion of C‐terminal 90 amino acids), ANAC005(1‐302)‐GFP (with deletion of C‐terminal 60 amino acids), ANAC005(1‐332)‐GFP (with deletion of C‐terminal 30 amino acids), and ANAC005(1‐342)‐GFP (with deletion of C‐terminal 20 amino acids) were all localized in the nucleus (Figure 2E–H). However, deletion of the C‐terminal 10 amino acids resulted in localization in both nucleus and plasma membrane (Figure 2I). These results show that C‐terminal 20 amino acids of ANAC005 play an important role in its localization of to the plasma membrane, whereas the N‐terminal region specifies nuclear localization.

### ANAC005 represses the growth of *Arabidopsis* and influences its morphogenesis

**Figure 3:**
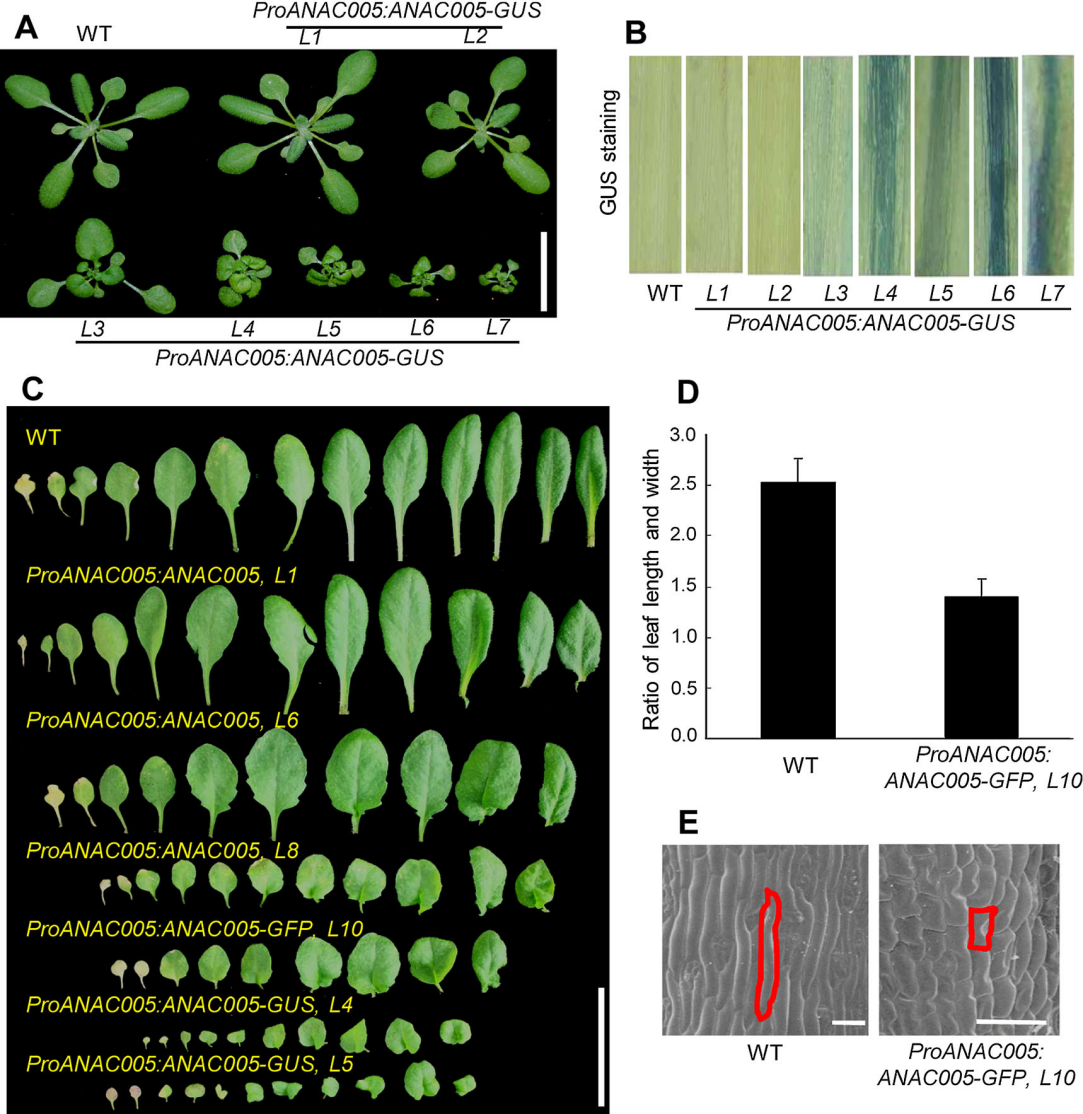
**Increasing ANAC005 expression caused dwarf phenotypes** **(A)** Three‐week‐old plants grown under long‐day conditions. Scale bar = 2cm. **(B)** GUS‐stained leaf petiole of 3‐week‐old plants grown in soil. **(C)** Comparison of rosette leaves. Leaves are arranged from the first leaf at the left to the latest leaf at the right. Scale bar = 2cm. **(D)** Ratio of leaf length and leaf width of wild type and *ProNAC005:NAC005‐GFP* transgenic plant grown under long‐day condition. **(E)** Cell length of leaf petiole in wild type and *ProNAC005:NAC005‐GFP* transgenic plants. Scale bar = 100 µm.

**Figure 4:**
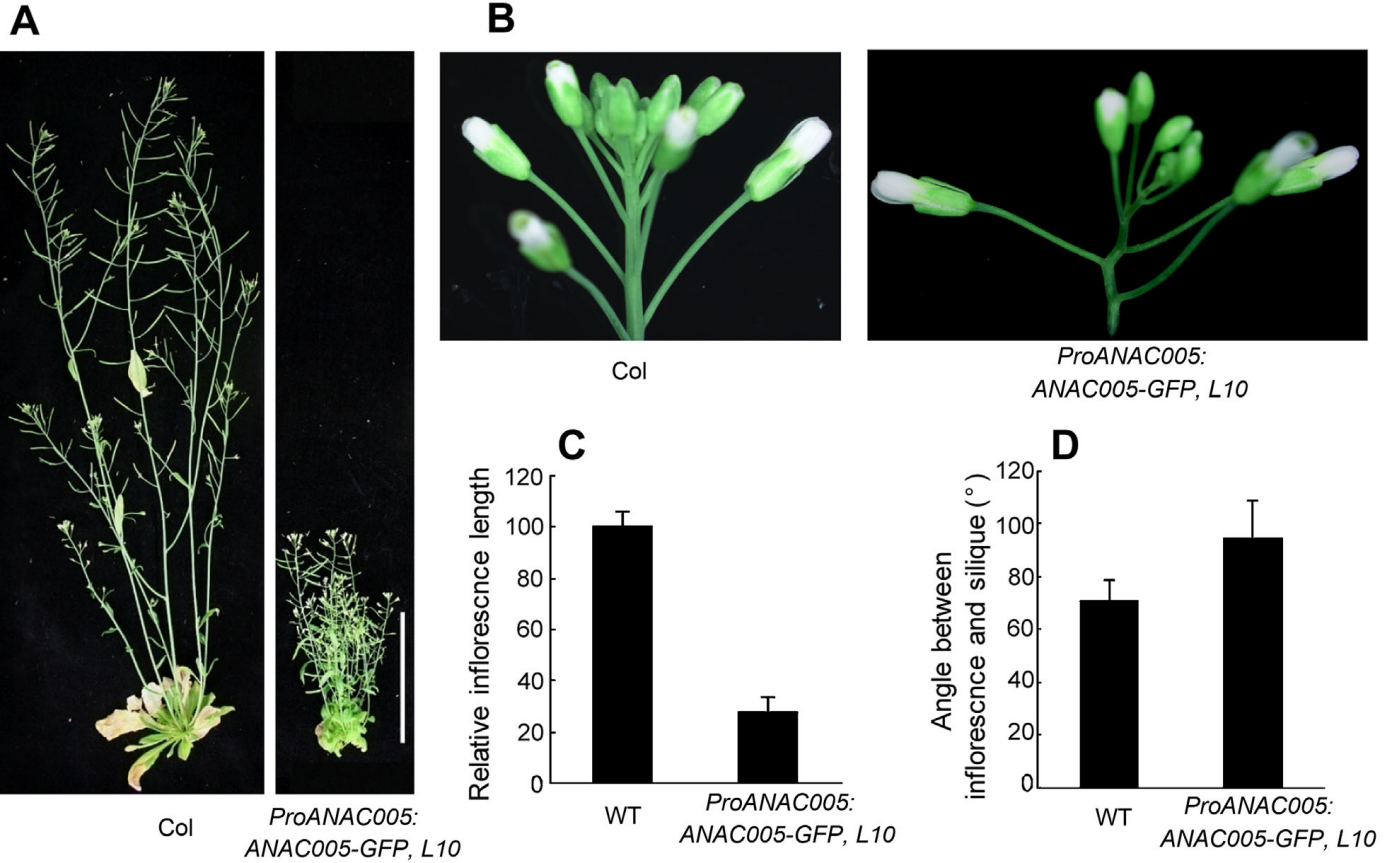
**Phenotypes of plants expressing *ANAC005‐GFP*** **(A)** 8‐week‐old plants grown under longer‐day conditions. Scale bar = 4 cm. **(B)** Inflorescence of plants grown under longer‐day conditions for 6 weeks. **(C)** Final height of the wild type and *ProANAC005:ANAC005‐GFP* transgenic plants. **(D)** Angle between inflorescence and silique stems.

The *proANAC005::ANAC005‐GUS* transgenic plants showed dwarf phenotypes with round rosette leaves, and the severity of phenotypes correlated with the intensities of GUS staining, indicating that expression of ANAC005‐GUS caused the dwarf phenotypes (Figure 3A, B). The tissue‐specific pattern of GUS staining, however, remained the same in lines that showed different levels of GUS and phenotype severity (Figures 3B, S3B). Similar dwarf phenotypes were observed in transgenic plants expressing the non‐fusion ANAC005 or ANAC005‐GFP fusion proteins, but the non‐fusion ANAC005 appeared to cause milder phenotypes compared to the GUS and GFP fusions (Figure 3C). Compared with wild type plants, the transgenic plants expressing high levels of *ANAC005* have shorter petiole and oval leaves (Figure 3C, D). Scanning electron microscopy (SEM) analysis showed the cell elongation is reduced in *ANAC005‐GFP* transgenic plants (Figure 3E). Furthermore, the ANAC005‐GFP transgenic plants show a bigger angle between inflorescence stem and pedicle (Figure 4A, C). When expressed using the CaMV 35S promoter, *ANAC005* causes similar dwarf phenotypes as driven by ANAC005 promoter (Figure S2).

We generated ANAC005 knockdown plants through artificial microRNA and RNA interference. But no phenotype was detected in the transgenic plant. With eight homologous genes in the subfamily (Ooka et al. 2003), ANAC005 is likely to have redundant functions with its homologs. We thus adopted the SRDX chimeric repressor method to study ANAC005 function (Hiratsu et al. 2003, 2004, 2002). Full‐length coding sequence of *ANAC005* was fused to the SRDX transcription repression domain (ANAC005‐SRDX) and expressed in transgenic *Arabidopsis* using the *CaMV 35S* promoter. To our surprise, the *35S:ANAC005‐SRDX* transgenic plants show no obvious morphological phenotype.

### ANAC005 inhibits xylem differentiation

**Figure 5:**
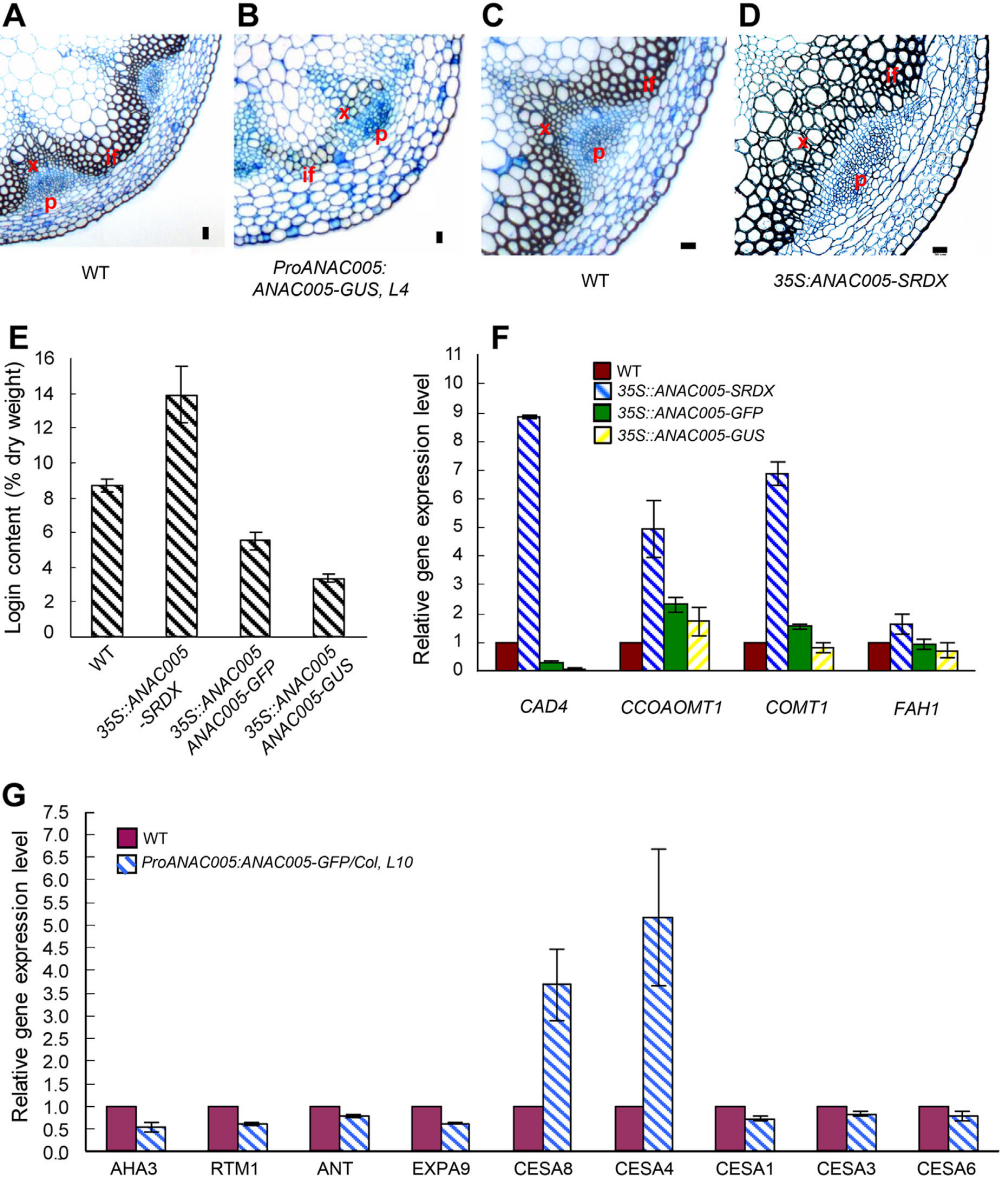
**ANAC005 inhibits xylem differentiation** **(A‐D)** Resin‐embedded transverse sections of the basal portion of the inflorescence stems of wild type **(A)** and *ProANAC005::ANAC005‐GUS* plants **(B)**, wild type **(C)** and 35*S:ANAC005‐SRDX* plants **(D)**. X, xylem; P, phleom; C, cambium; IF, interfascicular fiber. Scale bars = 20µm. **(E)** The lignin content of wild type (WT) and different transgenic plants. **(F, G)** Relative expression level of genes involved in lignin synthesis from 7‐day‐old plants **(F)** and genes related to vascular development from stem of 5‐week‐old plants **(G)** of wild type and transgenic plants.

Since ANAC005 is preferentially expressed in the vascular bundle, we investigated whether ANAC005 regulates vascular development. We performed sectioning and microscopic analysis of the influorescence stems of both *ProANAC005:ANAC005‐GUS* and 35S:ANAC005‐SRDX plants. As shown in Figure 5A–D, the *ProANAC005: ANAC005‐GUS* plants showed reduced xylem differentiation, whereas the *35S:ANAC005‐SRDX* plants showed increased xylem development. Consistent with the altered xylem development, the lignin content was increased in the *35S:ANAC005‐SRDX* plants but reduced in the *ProANAC005:ANAC005‐GUS* plants (Figure 5E).

We further analyzed vascular‐related genes in the inflorescence stem of the transgenic plants by qRT‐PCR. These genes include lignin synthesis genes (*CAD4, CCOAOMT1, COMT1, FAH1*), cambium marker (*EXPA9* and *ANT*) (Gray‐Mitsumune et al. 2004; Schrader et al. 2004), phloem marker (*AHA3* and *RTM1*) (DeWitt and Sussman 1995; Chisholm et al. 2001), primary cell wall cellulose synthesis genes (*CESA1*, *CESA3* and *CESA6*) (Desprez et al. 2007), and secondary cell wall cellulose synthesis genes (*CESA4* and *CESA8*) (Taylor et al. 2003). As shown in Figure 5F, CAD4 expression was highly increased in the *35S:ANAC005‐SRDX* plants, but decreased in two *ProANAC005: ANAC005‐GFP* lines, consistent with the alteration of lignin content and xylem development in these plants. Expression levels of *CCOAOMT1, COMT1, FAH1* were also increased in the *35S:ANAC005‐SRDX* plants, but not obviously altered in the *ProANAC005:ANAC005‐GFP* plants. All these results support that ANAC005 regulates lignin synthesis and xylem differentiation. Interestingly, genes related to cambium, phloem and primary cell wall were downregulated, while genes related to the secondary cell wall were upregulated in the *ProANAC005:ANAC005‐GFP* plants compared to wild type (Figure 5G).

## DISCUSSION

NAC proteins constitute a big family of plant‐specific transcription factors and function in diverse plant developmental processes (Ooka et al. 2003). In this article, we identified ANAC005 as a membrane‐bound NAC family transcription factor, and demonstrated its function in vascular tissue development.

Our results provide strong evidence that ANAC005 plays a role in vascular tissue development, specifically in controlling xylem development. First, previous microarray analysis (Zhao et al. 2005) and our promoter‐GUS reporter gene experiments showed preferential *ANAC005* expression in xylem. Our promoter‐GUS reporter gene also showed a low level expression in phloem cells. Second, expression of ANAC005‐GUS and the ANAC005‐SRDX dominant negative form reduced and increased, respectively, xylem development, lignin content, and expression of lignin biosynthetic gene *CAD4*. The opposite effects of ANAC005 and ANAC005‐SRDX suggest that ANAC005 regulates xylem development as a transcription activator and it reduces *CAD4* expression likely by activating a transcription repressor that inhibits *CAD4* expression. Overexpression of ANAC005‐GFP also increased the expression of CESA4 and CESA8, which are also activated by the NAC factor SND1, a major regulator of secondary wall synthesis (Zhong et al. 2008; Taylor‐Teeples et al. 2015). Recent studies showed that SND1 directly activates genes involved in secondary wall synthesis, but also activates KNAT7, which represses lignin synthetic genes (Taylor‐Teeples et al. 2015). ANAC005 may play a similar or overlapping role as SND1, in activating CESA4 and CESA8, but represses lignin synthesis through other transcription factors such as KNAT7. It is noteworthy that the dominant negative ANAC005‐SRDS and the *knat7* mutation promoted xylem development, whereas overexpression of ANAC005 had similar effects as overexpression of KNAT7 in repressing xylem development (Figure 5; Li et al. 2012). ANAC005‐SRDS and the *knat7* also had similar effects on lignin synthetic gene expression (Figure 5; Li et al. 2012). It will be interesting to test in future studies whether ANAC005 regulates lignin synthesis and xylem differentiation through KNAT7.

ANAC005 is a membrane‐associated NAC factor. The ANAC005‐GFP protein showed exclusive plasma membrane localization, which is mediated by the C‐terminal 20 amino acids of ANAC005. Deletion of the C‐terminal 10 amino acids partially disrupted ANAC005 localization at the membrane, whereas deletion of 20 amino acids completely abolished membrane localization, leading to accumulation in the nucleus. This C‐terminal sequence is highly conserved in several close homologs, such as ANAC003, ANAC004, and ANAC0048, but not in any of the known MTFs. Among the known NAC MTFs, NTM2/NTL13 showed the highest overall sequence homology to ANAC005, with 47% sequence identity in the amino acids 5‐288 region of ANAC005. Unlike the NTM/NTL factors, which have C‐terminal hydrophobic TM domain, the C‐terminal 20‐aa motif of ANAC005 contains eight basic amino acid residues and shares no homology with the C‐terminal TM motifs of NTM/NTL factors. We therefore conclude that ANAC005 is associated with membrane through a novel mechanism.

The distinct C‐terminal motifs suggest that the regulation of ANAC005 membrane disassociation is likely also different from the NTM/NTL factors. Indeed, salt treatment had no effect on ANAC005 localization (data not shown). The vascular phenotypes of ANAC005‐overexpressors suggest that ANAC005 membrane disassociation might be regulated developmentally by a cell type‐specific mechanism. Expression of ANAC005‐GFP and ANAC005‐GUS fusion proteins from the native ANAC005 promoter or overexpression of non‐fusion ANAC005, inhibited cell elongation and caused dwarf phenotypes (Figure 4). However, overexpression of ANAC005‐SRDX fusion affected xylem patterning, but did not cause severe dwarf phenotypes, suggesting that different transcriptional activities (activation vs repression) may be involved in ANAC005's effects on cell elongation and vascular development. Future study will be required to understand the mechanism that regulates ANAC005 membrane disassociation and activation, and to elucidate ANAC005's function in patterning vascular tissue differentiation.

## MATERIALS AND METHODS

### Plant material and growth

*Arabidopsis thaliana* ecotype Columbia‐0, and transgenic plants obtained in this study were grown at 22°C under white light (16‐h light/8‐h dark cycles) either on half‐strength MS medium or in the soil. *Arabidopsis* seeds were sterilized with 75% ethanol plus 0.01% Triton X‐100 for 15 min, then rinsed with 95% ethanol, and dried in the hood. The surface‐sterilized seeds were sown on 0.7% phytoagar plates containing half‐strength MS medium and 1% sucrose. The plates were kept at 4°C for 3 d and exposed to white light for 2 h before being transferred into the dark. Leaves, inflorescence stem, and silique were photographed and petioles, inflorescence stem, and angle between inflorescence and pedicle were measured using Image J software (http://rsb.info.nih.gov/ij/).

### Vector construction and transformation

To obtain the overexpression vector of *ANAC005*, a 1,609‐bp genomic fragment containing full‐length *ANAC005* open reading frame was amplified by PCR and then cloned into the BamHI and KpnI sites of the pSN1301 binary vector to place *ANAC005* under the control of the CaMV 35S promoter. The primer sequence used was 5′‐CGGGATCCATGGCGAATCCGGTGGGTTT‐3′ and 5′‐GGGGTACCTCATGTTCTTAGGTGAATTTTCTTGAC‐3′.

To get localized expression of ANAC005, 2,676‐bp/2,679‐bp (containing terminator codon) genomic segment containing promoter and gene sequence was amplified by PCR and ligated into pENTR^TM^/SD/D‐TOPO vector, and then ligated into pMDC163 and C3 by recombinant clone. The primer sequence used was 5′‐CACCAGTATCACAACTATGGGTCTGACT‐3′ and 5′‐TGTTCTTAGGTGAATTTTCTTGAC‐3′; 5′‐CACCAGTATCACAACTATGGGTCTGACT‐3′ and 5′‐CTATGTTCTTAGGTGAATTTTCTTGAC‐3′.

The 35S:ANAC005‐GFP, 35S:ANAC005(1‐272)‐GFP, 35S:ANAC005(1‐302)‐GFP, 35S:ANAC005(1‐332)‐GFP, 35S:ANAC005(1‐342)‐GFP, and 35S:ANAC005(1‐352)‐GFP fusion construct was generated by inserting a full‐length *ANAC005* cDNA without stop codon, a 816 bp cDNA segment without 270 bp segment in the 3′ terminal, a 906 bp cDNA segment without 180 bp cDNA segment in the 3′ terminal, a 996 bp cDNA segment without 90 bp segment in the 3′ terminal, a 1,026 bp cDNA segment without 60 bp in the 3′ terminal, or a 1,056 bp cDNA segment without 30 bp in the 3′ terminal into pENTR^TM^/SD/D‐TOPO vector, and then ligated into pMDC83 by recombinant clone. The forward primer was 5′‐CACCATGGCGAATCCGGTGGGTTT‐3′ and the reverse primers were 5′‐TGTTCTTAGGTGAATTTTCTTGAC‐3′, 5′‐TGTATCATCCTGTGAAAGAAAC‐3′, 5′‐TTTGTTCTTGATCGTCTCTTGAC‐3′, 5′‐ATGCTCACCAATCTCAGTCCCTTG‐3′, 5′‐TGAGTTAGGAGATTCCTGCAAG‐3′, and 5′‐AGTTGTTGCAGAAATCGGATCAG‐3′.

To knock down *ANAC005* in *Arabidopsis* using RNAi, a specific sequence was amplified from wild type *Arabidopsis* genomic DNA with primers 5′‐GGGGTACCACTAGTAACACGAGCCATGTCGATG‐3′ and 5′‐CGGGATCCGAGCTCATGTTCTTAGGTGAATTTTCTTG‐3′. This fragment was first digested by BamHI and KpnI for the reverse insert to vector pTCK309. The forward insert was generated by SpeI and SacI digestion.

To get the dominant repressor of ANAC005, the full‐length cDNA sequence was amplified with primers 5′‐CGGGATCCATGGCGAATCCGGTGGGTTT‐3′ and 5′‐GGACTAGTTGTTCTTAGGTGAATTTTCTTGAC‐3′. Then the segment is ligated into p35SSRDX vector between BamHI and SpeI sites.

The constructs were transformed into the Agrobacterium tumefactions strain GV3101 and then introduced into *Arabidopsis thaliana* plants via floral dip method.

### Total RNA extraction and quantitative RT‐PCR analysis

Total RNA was extracted using the Trizol reagent (Invitrogen, Carlsbad, California, USA). About 500 ng RNA was reverse‐transcribed by AMV reverse transcriptase (Takara Biotechnology, Dalian, China) following the manufacture's instruction. Quantitative RT‐PCR analyses were carried out on ABI7500 (Applied Biosystems, Foster City, California, USA) by using SYBR Green reagent (Toyobo, Osaka, Japan). Three biological repeats and three technical repeats were performed in each treatment. The *UBC30* gene was used as internal reference for all the qRT‐PCR analysis.

### Assays of GUS activity

The histochemical GUS assays were performed in a staining solution containing 0.5 mg/mL 5‐bromo‐4‐chloro‐3‐indolyl glucuronide (X‐Gluc) in 0.1 M Na_2_HPO_4_, pH 7.0, 10 mM Na_2_EDTA, 0.5 mM potassium ferricyanide/ ferrocyanide, and 0.06% Triton X‐100 (Jefferson et al. 1987). Samples were infiltrated under vacuum for 10 min and then incubated at 37°C overnight. The staining buffer was removed, and the samples were cleared in 70% ethanol. All observations by light microscopy were made with the Olympus BX51 microscope system.

### Semi‐thin section and microscopy

*Arabidopsis* stems were fixed overnight in FAA buffer (3.7% formalin, 5% acetic acid, 50% alcohol). Samples were embedded in Spurr's resin (SPI‐CHEM) and sectioned to about 0.5ìm. The microsections were stained with 0.05% toluidine blue‐O (Sigma‐Aldrich) before imaging under a microscope (Zeiss Imager M2).

### Accession numbers

Sequence data from this article can be found in the *Arabidopsis* Genome Initiative or GenBank/EMBL databases under the following accession numbers: *ANAC005* (*At1g02250*), *APL* (*At1g79430*), *RTM1* (*At1g05760*), *AHA3* (*At5g57350*), *ANT* (*At4g37750*), *EXPA9* (*At5g02260*), *CESA8* (*At4g18780*), *CESA4* (*At5g44030*), *CESA1 (At4g32410),* *CESA3 (At5g05170)* and *CESA6* (*At5G64740*).

## AUTHOR CONTRIBUTIONS

J.Z., Z.Y.W. and S.W.Z. designed the study, analyzed the data, and wrote the article. J.S.L and F.N.M. performed gene, protein expression analysis and physiological analysis. Z.Z.Z. and H.L. performed transgenic experiment and subcellular localization of proteins. W.H.L. and X.M.L. assisted in physiological analysis. J.Z. performed all the other studies.

## Supporting information

Additional supporting information may be found in the online version of this article at the publisher's web‐site.

**Figure S1.** ANAC005 is a classic NAC family protein **(A)** Structure analysis of predicted amino acid sequence of ANAC005. The yellow, light green, light blue, red, and dark green box indicate the motif of A to E of NAC domain. The gray box indicates the TAR (Transcription activation region) motif. The underline marker sequence is a predicated transmembrane motif (TM) according to the TopPred‐v2 program. **(B)** Sequence alignment of the predicted amino acids of ANAC005 and closely related members of NAC proteins. Shade residues indicate positions where amino acid are highly conserved. The yellow, light green, light blue, red, and dark green overlines indicate the motif of A to E of NAC domain. The gray line indicates the TAR motif. The black line indicates C‐terminal 20 amino acids of ANAC005, ANAC003, ANAC004 and ANAC048.
**Figure S2.** Overexpression of *ANAC005* causes dwarf phenotypes Phenotypes (upper panel) and semiquantitative RT‐PCR analysis of *ANAC005* expression (lower panel) of wild type (WT) and different lines of *35S::ANAC005. UBC30* was used as a control. Scale bar = 5 cm.
**Figure S3.** *ANAC005* is expressed preferentially in vascular bundles **(A–C)** GUS‐stained 3‐day‐old seedlings of wild type **(A)**, *ProANAC005::ANAC005‐GUS* plants without phenotype **(B)** and weak phenotype **(C)**. Scale bar = 1 mm.
