# Supplementary material for: ANAC005 is a membrane‐associated transcription factor and regulates vascular development in Arabidopsis
Source: J Integr Plant Biol. 2015 Sep 25;58(5):442–51. doi: 10.1111/jipb.12379 (PMC5054944; doi:10.1111/jipb.12379)
Supplement: Supplementary file 1 — Figure S1. ANAC005 is a classic NAC family protein (A) Structure analysis of predicted amino acid sequence of ANAC005. The yellow, light green, light blue, red, and dark green box indicate the motif of A to E of NAC domain. The gray box indicates the TAR (Transcription activation region) motif. The underline marker sequence is a predicated transmembrane motif (TM) according to the TopPred‐v2 program. (B) Sequence alignment of the predicted amino acids of ANAC005 and closely related members of NAC proteins. Shade residues indicate positions where amino acid are highly conserved. The yellow, light green, light blue, red, and dark green overlines indicate the motif of A to E of NAC domain. The gray line indicates the TAR motif. The black line indicates C‐terminal 20 amino acids of ANAC005, ANAC003, ANAC004 and ANAC048. Figure S2. Overexpression of ANAC005 causes dwarf phenotypes Phenotypes (upper panel) and semiquantitative RT‐PCR analysis of ANAC005 expression (lower panel) of wild type (WT) and different lines of 35S::ANAC005. UBC30 was used as a control. Scale bar = 5 cm. Figure S3. ANAC005 is expressed preferentially in vascular bundles (A–C) GUS‐stained 3‐day‐old seedlings of wild type (A), ProANAC005::ANAC005‐GUS plants without phenotype (B) and weak phenotype (C). Scale bar = 1 mm. [file JIPB-58-442-s001.ppt]

## Slide 1
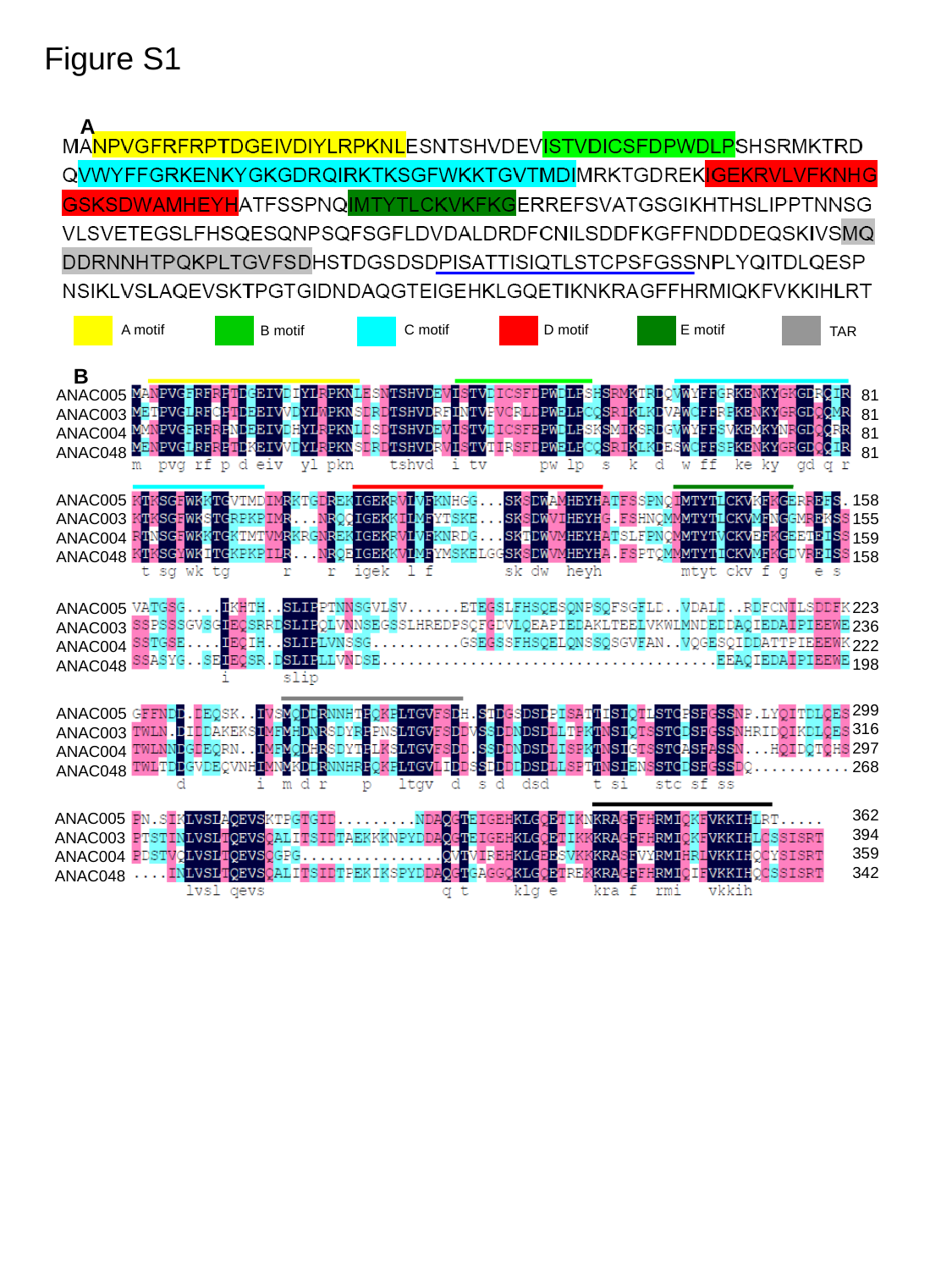

Figure S1
A
 A motif
 C motif
 D motif
 E motif
 B motif
 TAR
B
ANAC005
ANAC003
ANAC004
ANAC048
81
81
81
81
ANAC005
ANAC003
ANAC004
ANAC048
158
155
159
158
223
236
222
198
ANAC005
ANAC003
ANAC004
ANAC048
299
316
297
268
ANAC005
ANAC003
ANAC004
ANAC048
362
394
359
342
ANAC005
ANAC003
ANAC004
ANAC048

## Slide 2
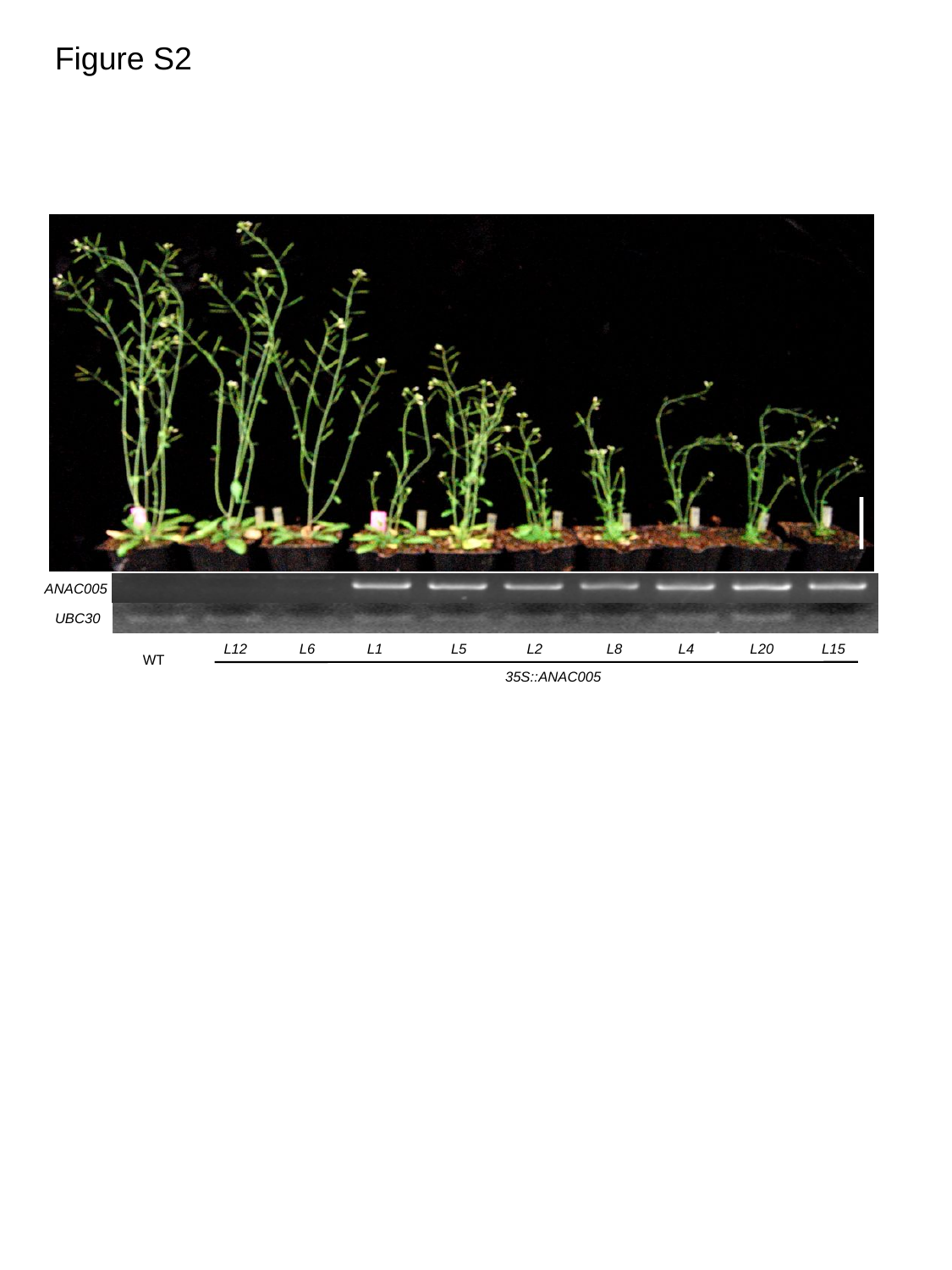

Figure S2
ANAC005
UBC30
 L12 L6 L1 L5 L2 L8 L4 L20 L15
WT
35S::ANAC005

## Slide 3
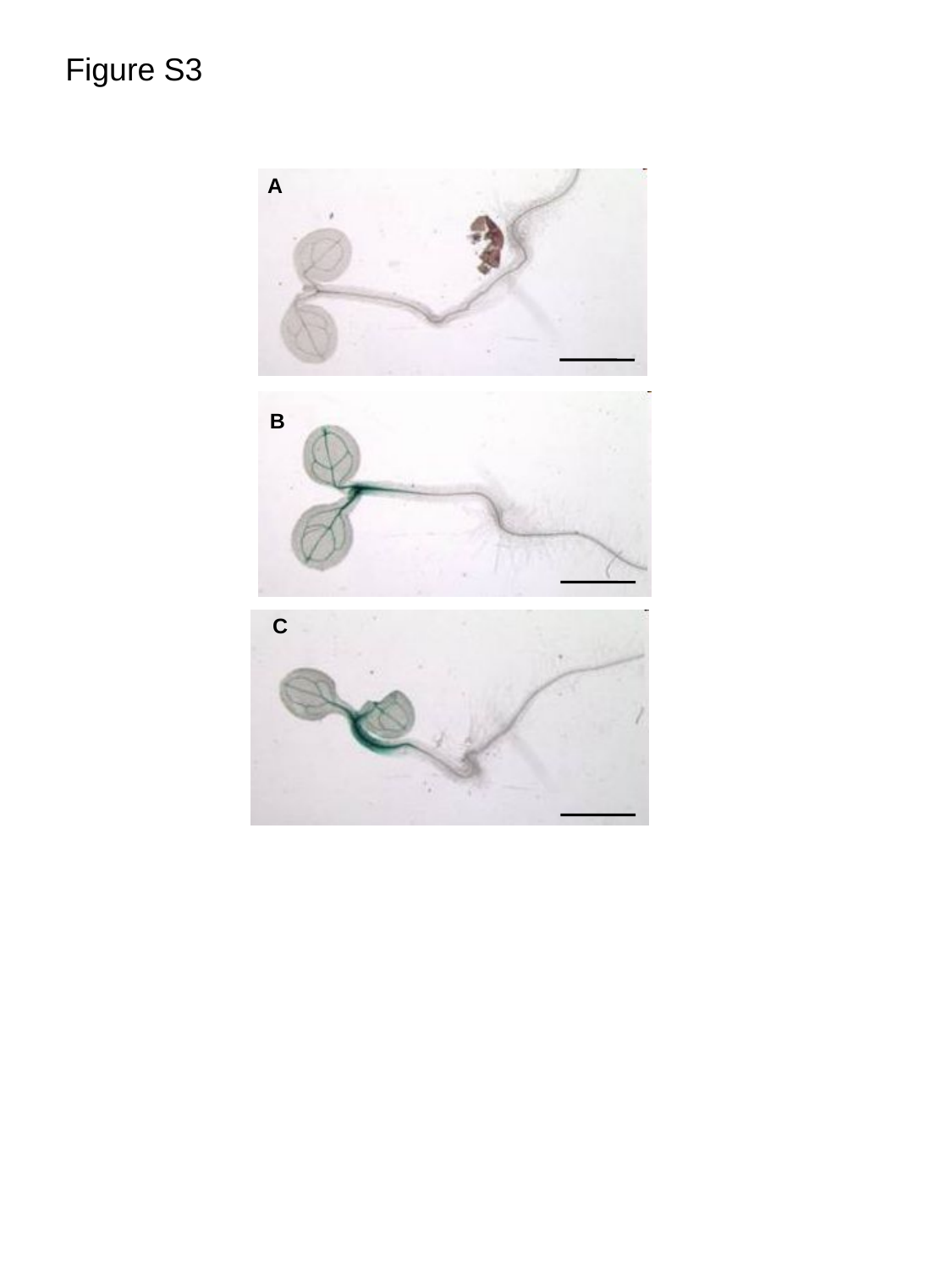

Figure S3
A
B
C
